# Supplementary material for: Accelerated DNA replication fork speed due to loss of R-loops in myelodysplastic syndromes with SF3B1 mutation
Source: Nat Commun. 2024 Apr 8;15:3016. doi: 10.1038/s41467-024-46547-7 (PMC11001894; doi:10.1038/s41467-024-46547-7)
Supplement: Supplementary file 1 — Supplementary Information [file 41467_2024_46547_MOESM1_ESM.pdf]

Supplementary Informations

Supplementary Figures

Supplementary Methods

Supplementary Fig. 1: RNA-sequencing data of splicing factor-mutated bone marrow mononuclear cells.

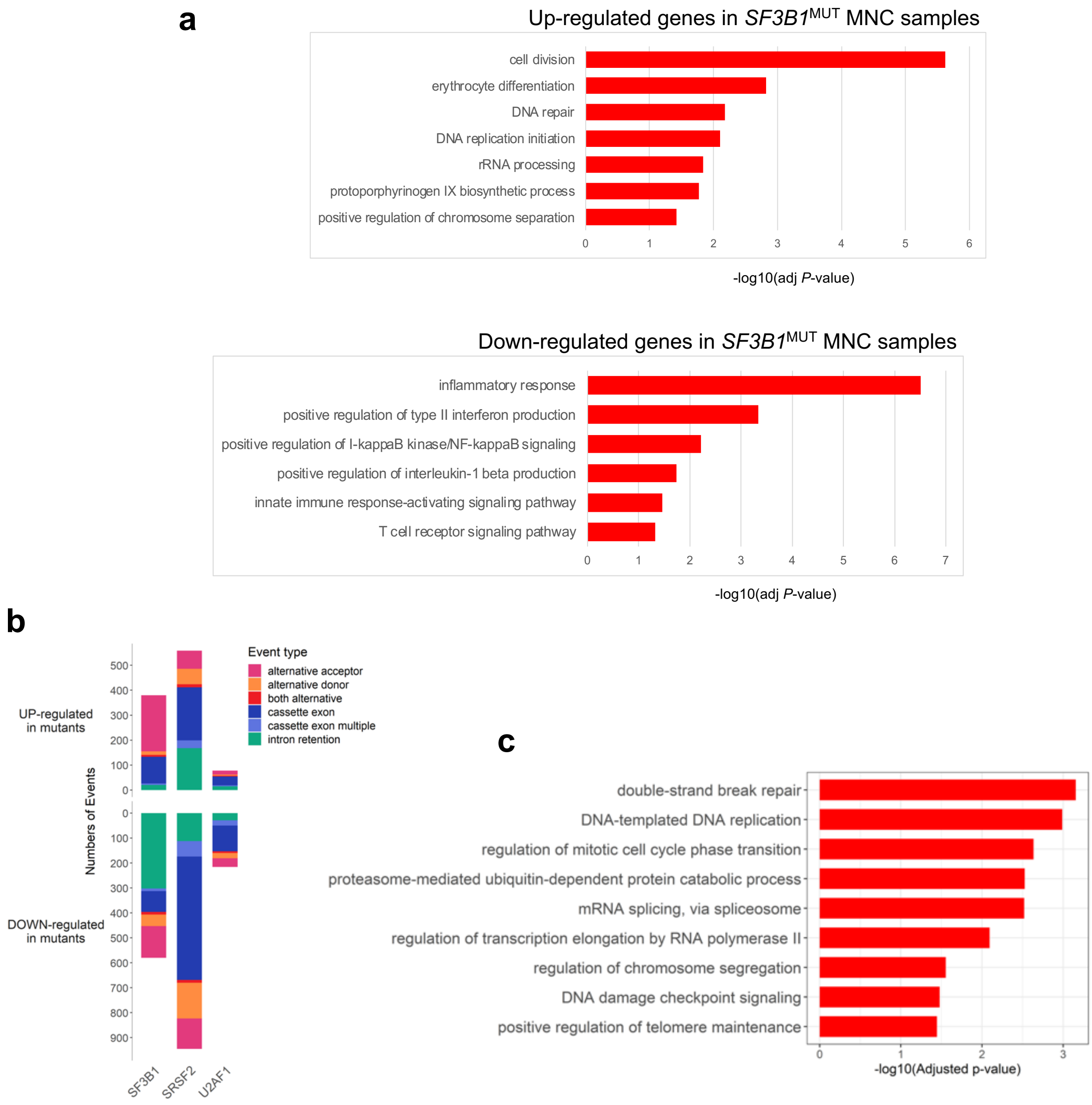

a. Gene ontology analysis of up- or down-regulated genes in 21 *SF3B1*^{MUT} and 6 *SF3B1*^{WT} (4 *SRSF2*^{MUT} and 2 *SF*^{WT}) MNC samples. **b.** Splicing events of 74 *SF3B1*^{MUT}, 30 *SRSF2*^{MUT} or 11 *U2AF1*^{MUT} MNC samples were identified in comparison with 70 triple-negative samples. Barplots showing the splicing events related to each mutation. The bars over 0 indicate the events the most frequently retrieved in mutant cases and the bars under 0 indicate the events the most frequently retrieved in wild-type cases. **c.** Gene ontology over-representation analysis of 822 genes affected by 1,027 intron retention reductions in 21 *SF3B1*^{MUT} and 6 *SF3B1*^{WT} (4 *SRSF2*^{MUT} and 2 *SF*^{WT}) MNC samples. MNC: mononuclear cells.

Supplementary Fig. 2: Proliferation and apoptosis features of human primary *SF3B1*^{MUT} erythroblasts.

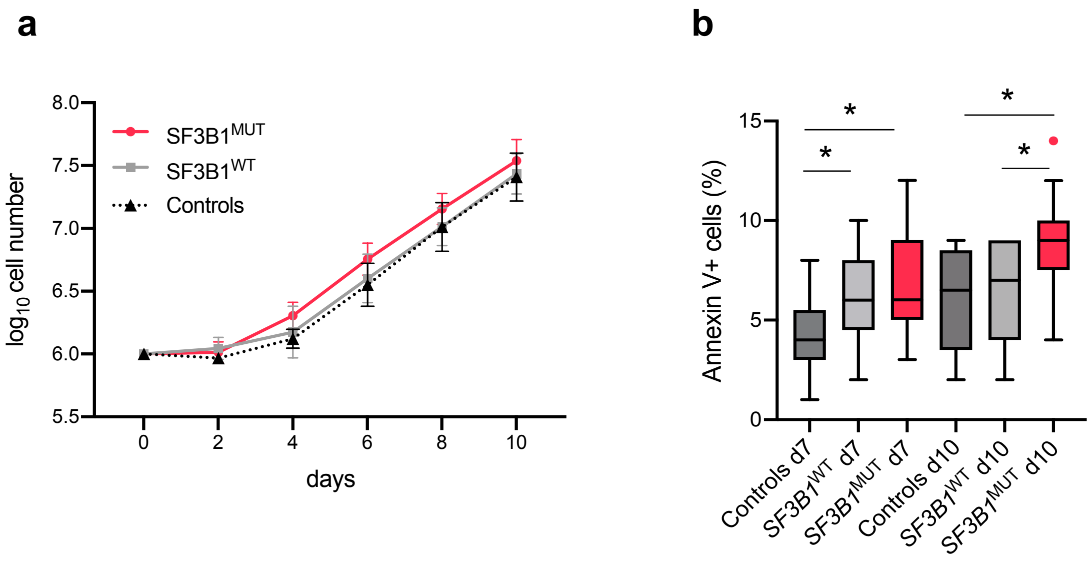

a. Cell proliferation. Results are expressed as log10 (cell number) ± standard error of the mean.
b. Apoptosis. Results are expressed as medians of percentages of annexinV-positive cells. Boxes representing interquartile ranges and bars representing minimum and maximum values are shown.

Supplementary Fig. 3: Pathway analysis of transcriptomic data from human basophilic and polychromatophilic erythroblasts

a

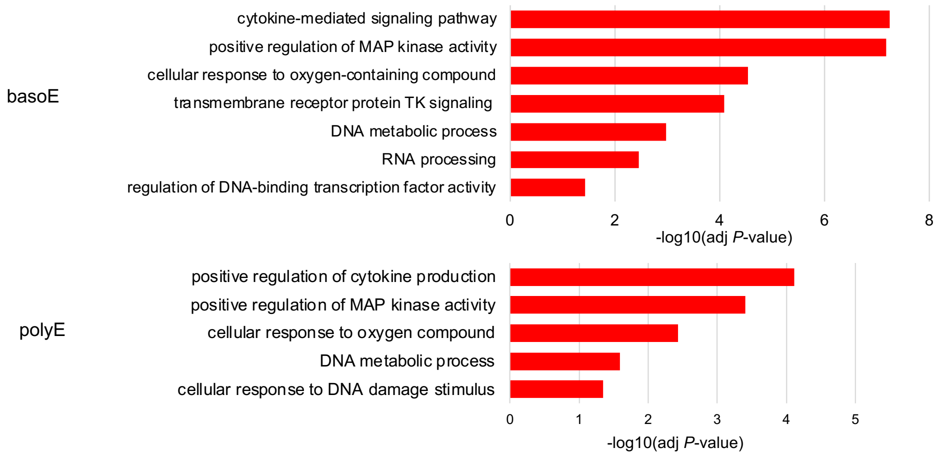

b

	GS follow link to MSigDB	GS DETAILS	SIZE	ES	NES	NOM p-val	FDR q-val	FWER p-val	RANK AT MAX
1	KEGG DNA REPLICATION	Details ...	35	-0.55	-1.81	0.000	0.019	0.010	6356
2	REACTOME BER	Details ...	43	-0.48	-1.66	0.005	0.011	0.005	7097
3	G1 - S PHASES CHECKPOINT	Details ...	65	-0.39	-1.47	0.017	0.120	0.177	8685
4	KEGG NUCLEOTIDE EXCISION REPAIR	Details ...	43	-0.38	-1.31	0.080	0.239	0.418	6356
5	KEGG MISMATCH REPAIR	Details ...	22	-0.36	-1.07	0.370	0.583	0.812	8697
6	CELL CYCLE	Details ...	122	-0.25	-1.05	0.332	0.542	0.847	5490
7	GOBP_DNA REPAIR (598)	Details ...	536	-0.19	-0.96	0.710	0.688	0.942	6061
8	REACTOME HR	Details ...	65	-0.27	-1.05	0.351	0.377	0.401	6356
9	REACTOME NHEJ	Details ...	30	0.42	1.26	0.166	0.137	0.228	8654

c

	GS follow link to MSigDB	GS DETAILS	SIZE	ES	NES	NOM p-val	FDR q-val	FWER p-val	RANK AT MAX
1	KEGG NHEJ	Details ...	10	-0.50	-1.25	0.201	1.000	0.597	1443
2	CELL CYCLE	Details ...	122	-0.25	-1.08	0.259	1.000	0.873	8082
3	KEGG DNA REPLICATION	Details ...	34	-0.28	-0.96	0.511	1.000	0.972	11658
4	GOBP_DNA REPAIR (598)	Details ...	532	-0.18	-0.94	0.712	1.000	0.978	8625
5	KEGG HR	Details ...	24	-0.29	-0.91	0.588	1.000	0.984	10516
6	KEGG NUCLEOTIDE EXCISION REPAIR	Details ...	42	-0.25	-0.91	0.605	1.000	0.984	7130
7	KEGG BASE EXCISION REPAIR	Details ...	33	-0.26	-0.88	0.630	0.939	0.991	7736
8	G1 - S PHASES CHECKPOINT	Details ...	64	-0.20	-0.76	0.884	1.000	0.999	4278
9	ATR BRCA1 PATHWAY	Details ...	20	-0.23	-0.69	0.878	1.000	1.000	1865
10	KEGG MISMATCH REPAIR	Details ...	21	-0.21	-0.64	0.937	0.955	1.000	8083

a. Gene Ontology (GO) over-representation analysis of upregulated genesets of *SF3B1*^{MUT} erythroblasts. Upper panel: basophilic erythroblasts (basoE). Bottom panel: polychromatophilic erythroblasts (polyE). No significant GO terms appeared in the downregulated genesets. **b.** Geneset enrichment analysis (GSEA) of up- and down-regulated transcripts in *SF3B1*^{MUT} basoE compared to wildtype basoE. **c.** GSEA of up- and down-regulated transcripts in *SF3B1*^{MUT} polyE compared to wildtype polyE.

Supplementary Fig. 4: Comparison of the proteome of *SF3B1*^{MUT} versus *SF3B1*^{WT} primary human erythroblasts.

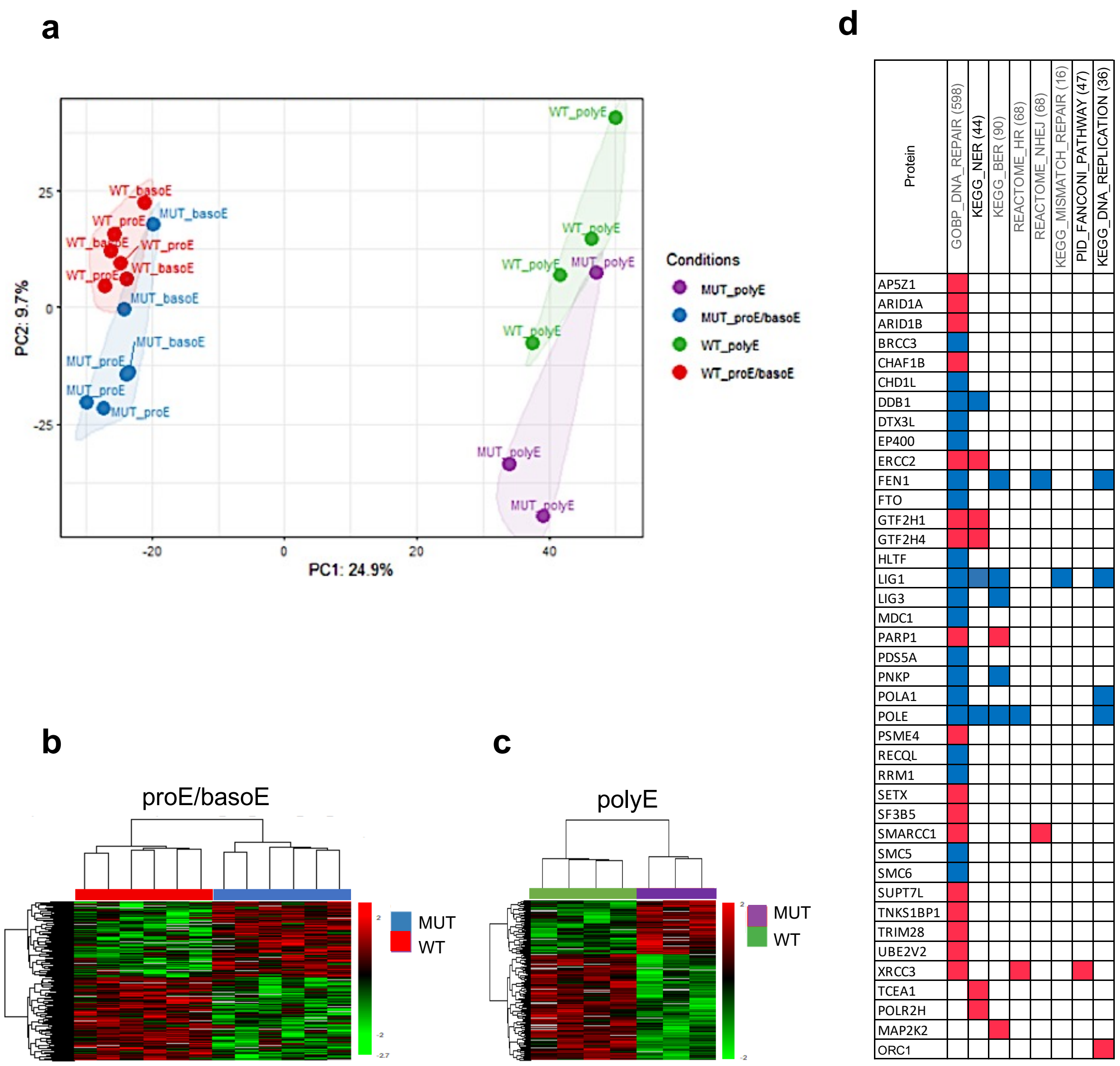

a. Principal component analysis based on the z-score of Log$_2$(label free quantification (LFQ) intensity) of the proteins with at least 70% of values in at least one condition. **b-c**. Heatmaps of the z-scores of LFQ of each differential protein (LFQ intensity minus the mean of all samples divided by their standard deviation). The more z-score is high, the more the protein is expressed in this sample compared to the others. **b**. Proerythroblasts (proE) and basophilic erythroblasts (basoE). **c**. Polychromatophilic erythroblasts (polyE). **d.** Visualization of up- and down-regulated proteins of the DNA repair and DNA replication pathways in the proteome of basophilic erythroblasts (red squares: upregulated proteins; blue square: downregulated proteins).

Supplementary Fig. 5: DNA:RNA immunoprecipitation (DRIP)-sequencing of human primary erythroblasts.

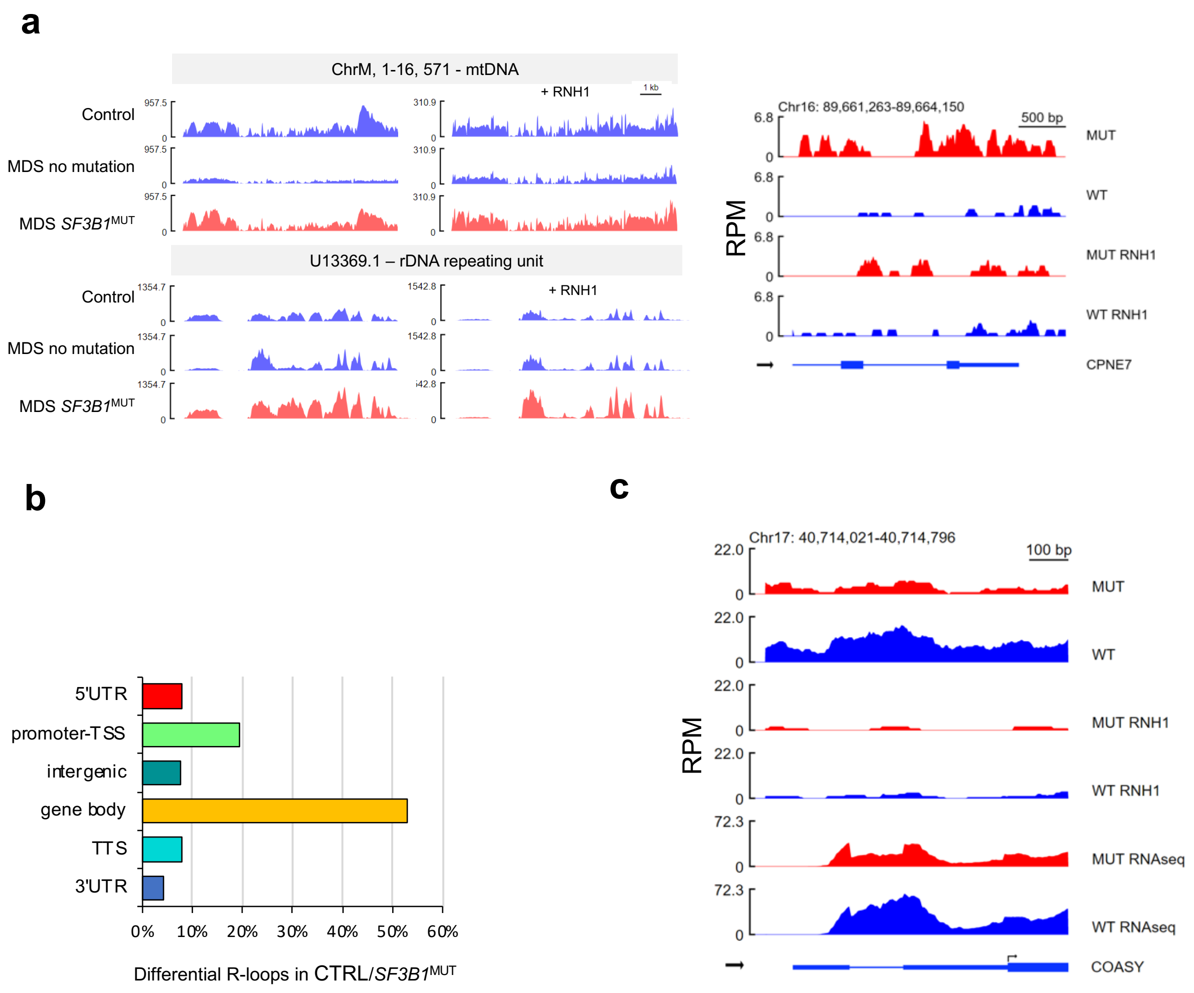

a. Examples of R-loops expressed as reads per million (RPM) at specific loci showing elevated R-loops at mitochondrial DNA, or rDNA (left panel) and increase of R-loops near the TTS of *CPNE7* gene (right panel) in one *SF3B1*^{MUT} sample compared to a control and one *SF*^{WT} sample. Peak specificity was assessed by treatment with RNaseH1 (RNH1). **b.** Distribution of differential R-loops observed in controls and absent in *SF3B1*^{MUT} samples according to gene features. **c.** Overlap of DRIP-seq and RNA-seq analyses at *COASY* locus. Differential R-loop and intron retention reduction in *SF*^{WT} versus *SF3B1*^{MUT} erythroblasts are shown. Gene structures (Gencode GRCh37) are indicated. RPM: reads per million

Supplementary Fig. 6: DNA:RNA immunoprecipitation (DRIP)-qPCR at specific loci.

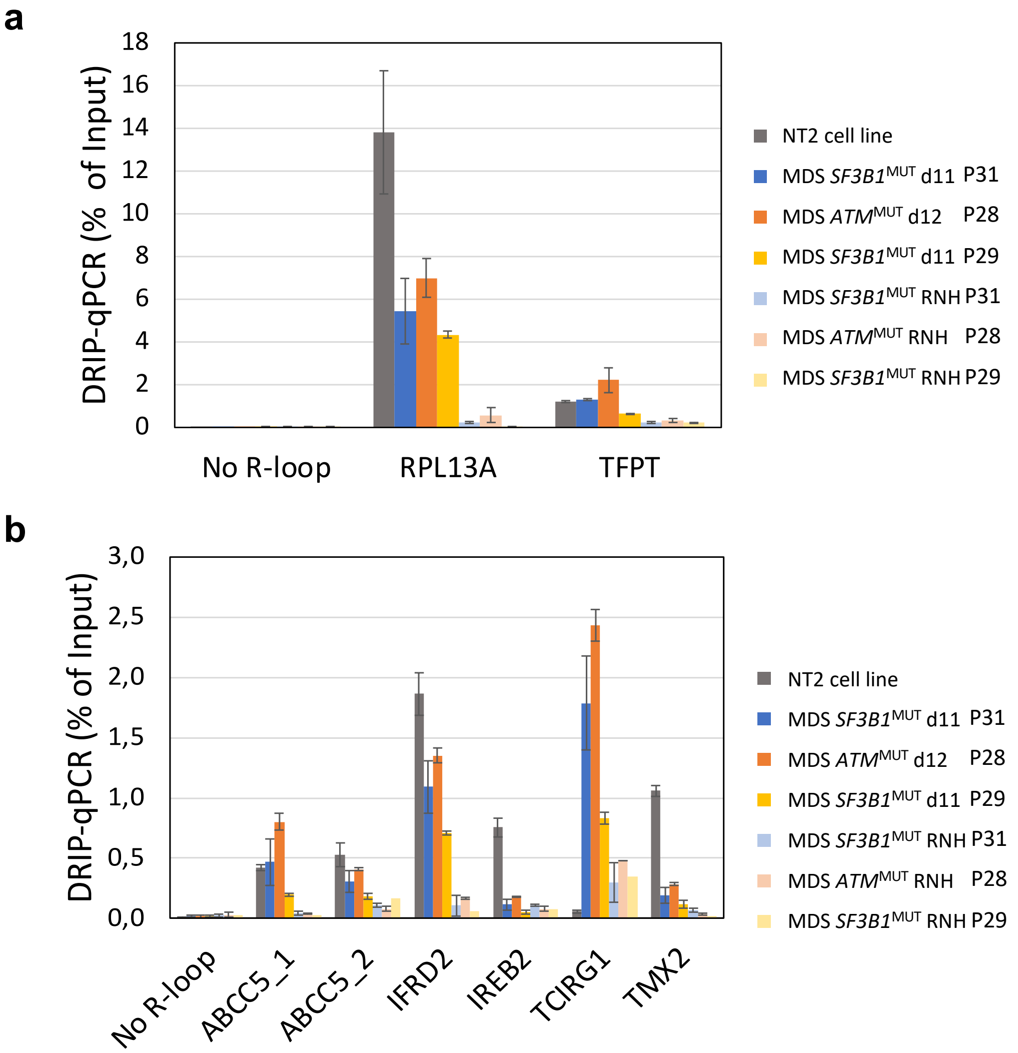

MDS erythroblast samples (2 with *SF3B1* mutation (P29, P31) and 1 with *ATM* mutation (P28) and NT2 cell line as positive control were used. **a.** Bar chart of DRIP-qPCR (as percent input) for one negative control locus (corresponds to an intergenic region ~200kb downstream of *DNAJB1*) and two positive R-loop forming loci (*RPL13A* and *TFPT*). Each bar is the average of IPs performed. Error bars represent standard error. **b.** Bar chart of DRIP-qPCR (as percent input) for one negative control locus (corresponds to an intergenic region ~200kb downstream of *DNAJB1*) and 5 specific loci: 2 at genes with an intron retention reduction (*ABCC5, TCIRG1*) and 3 genes without IRR (*IFRD2, IREB2, TMX2*) in *SF3B1* mutant condition. Each bar is the average of IPs performed. Error bars represent standard error. Controls with RNase H (RNH) pretreatment are shown.

Supplementary Fig. 7: Single stranded DNA exposure in *SF3B1*^{MUT} erythroblasts

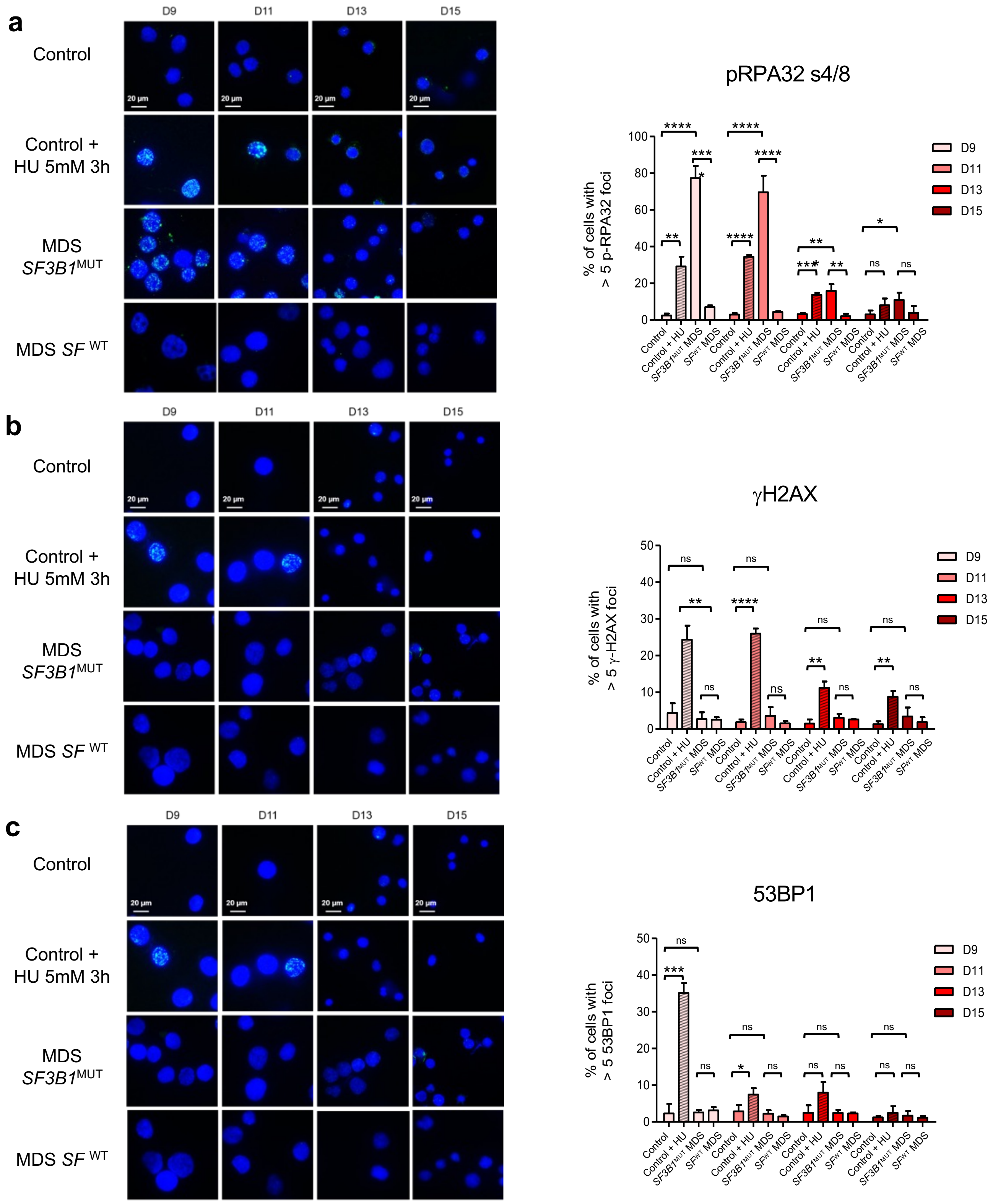

Erythroblasts derived from 5 *SF3B1*^{MUT} MDS, 3 splicing factor (*SF*)^{WT} MDS and 3 healthy donors as controls treated or not with 5mM HU for 3h, were harvested at days 9, 11, 13 and 15 of cultures, and labelled using specific antibodies to pRPA32 s4/s8 (**a**), γH2AX (**b**), and 53BP1 (**c**). Images were acquired at 100X magnification (scale: 20 µm). Cells with > 5 intranuclear foci were considered positive. Results are expressed as percentages of positive cells relative to counted cells. Mann-Whitney tests; * $P < 0.05$; ** $P < 0.01$; *** $P < 0.001$; **** $P < 0.0001$.

Supplementary Fig. 8: Phenotypic characterization of murine G1E-ER4 Crispr-Cas9 *Sf3b1*$^{K700E/+}$ erythroblasts.

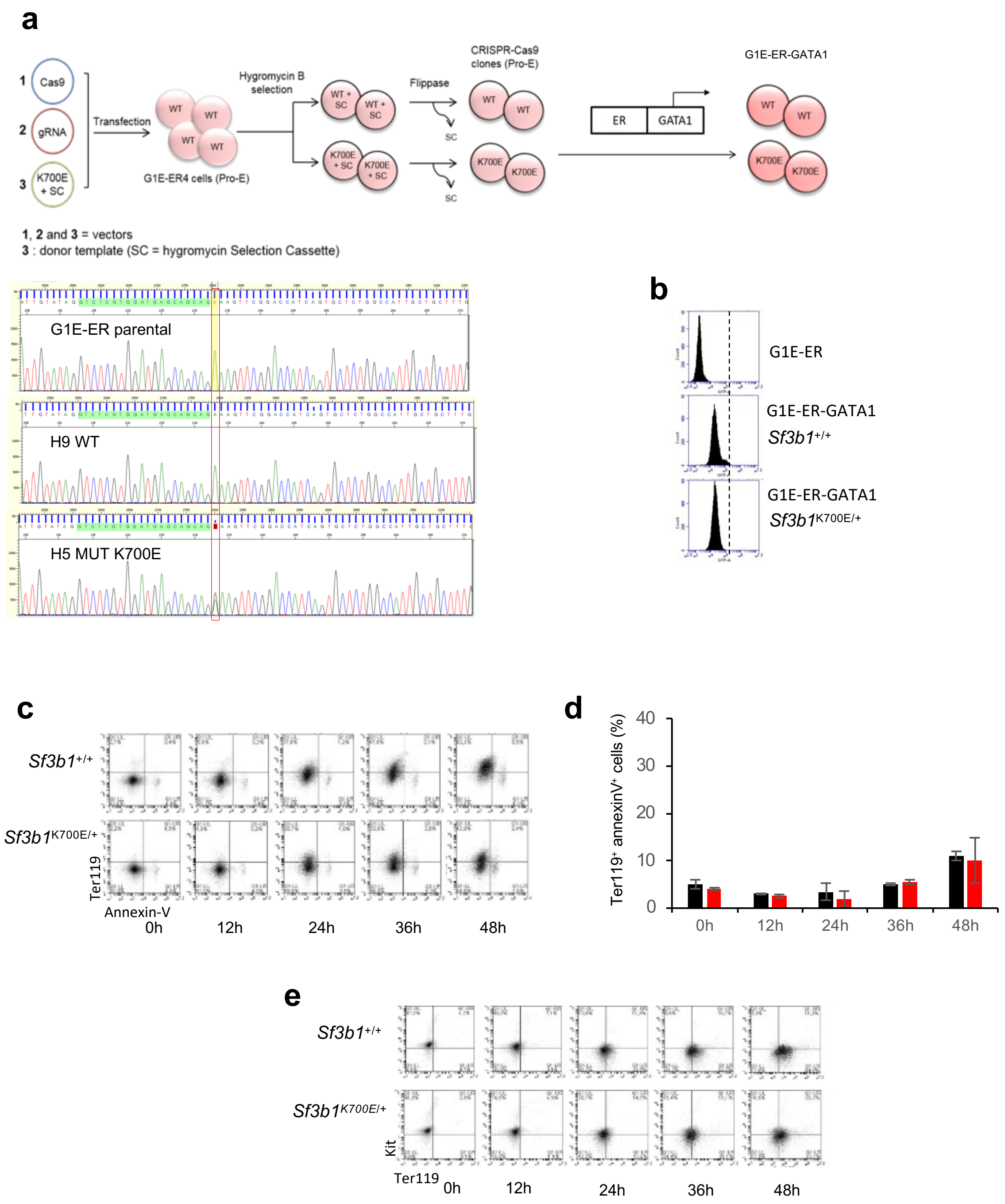

a. Experimental design of Crispr-Cas9 strategy and transfection of ER-GATA1 sequence using a GFP-expressing vector in G1E-ER4 cells. **b.** Expression of GATA1 by flow cytometry measurement of GFP^{+} cells. **c.** Apoptosis of *Sf3b1*$^{K700E/+}$ and *Sf3b1*$^{+/+}$ clones upon estradiol treatment for 0 to 48h by labelling the cells with Ter119 and Annexin V. Flow cytometry scatter plots representative of 3 independent experiments. **d**. Quantification of Ter119^{+} Annexin V^{+} cells expressed as means ± SEM of 3 experiments. **e.** Scatter plots of flow cytometry with Ter119 and Kit (CD117) antibodies representative of 3 independent experiments.

Supplementary Fig. 9: Transcriptomic and metabolomic analyses of murine G1E-ER4 *Sf3b1*$^{K700E/+}$ and *Sf3b1*$^{+/+}$ cells before and after induction of differentiation with estradiol.

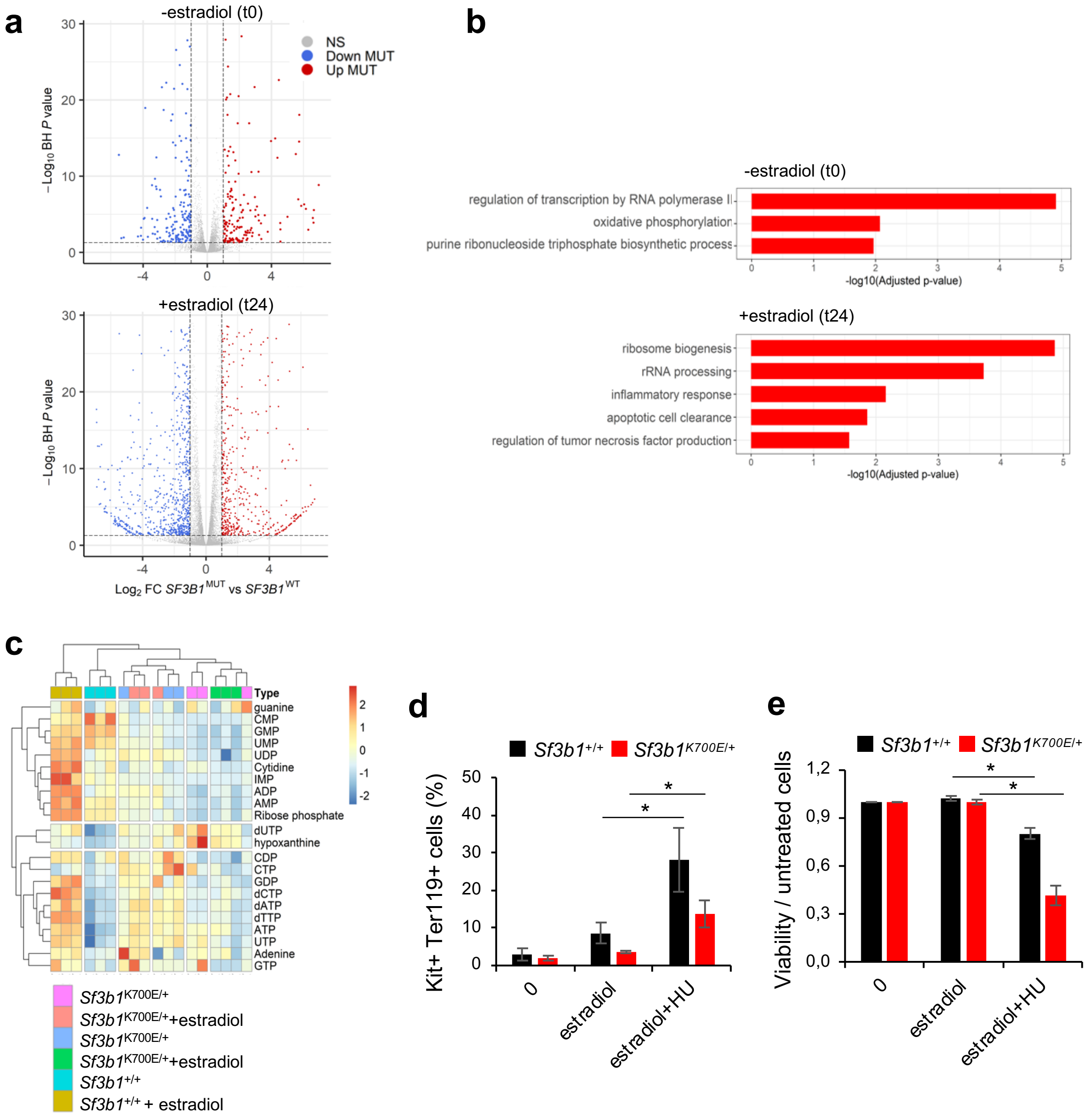

a. Volcano plots of up- and downregulated genes in *Sf3b1*$^{K700E/+}$ at t0 before and t24 after induction of differentiation with estradiol with log2 fold change (FC) > |1| and Benjamini-Hochberg (BH)-adjusted *P*-value < 0.05. **b.** Barplots representing Gene Ontology enrichment analysis of up- and down-regulated genes in *Sf3b1*$^{K700E/+}$ compared to *Sf3b1*$^{+/+}$ cells at t0 (-estradiol) and t24 (+estradiol) with log2 fold change (FC) > |1| and Benjamini-Hochberg-adjusted *P*-value < 0.05. **c.** Heatmap representing unsupervised clustering of samples according to signal intensities of the metabolomic data showing variations of the amounts of purines, pyrimidines, monophosphate nucleotides, diphosphate nucleotides, NTP, dATP, dTTP and dCTP in *Sf3b1*$^{K700E/+}$ erythroblasts compared to *Sf3b1*$^{+/+}$ erythroblasts induced or not in differentiation with estradiol for 24h. **d, e.** Effect of hydroxyurea (HU) at 0.2 mM for the last 16h of the 24h-treatment with estradiol on the differentiation (Ter119/Kit) and viability (FSC/SSC) of *Sf3b1*$^{K700E/+}$ and *Sf3b1*$^{+/+}$ clones evaluated by flow cytometry.

Supplementary Fig. 10: **Effects on HDACi on R-loop profiles near gene promoters.**

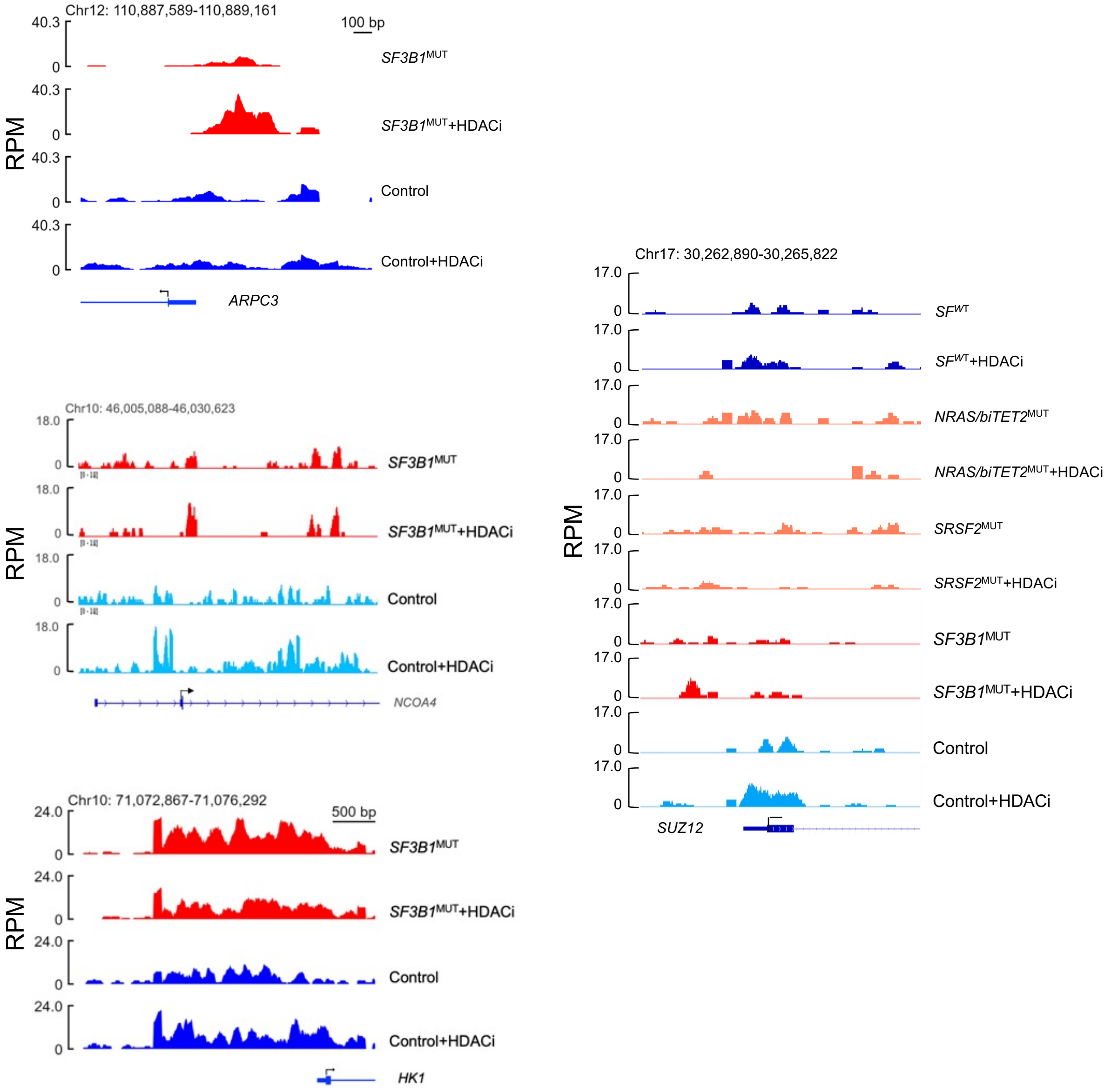

SF3B1^{MUT}, *SF3B1*^{WT} and control erythroblasts were treated with HDACi 0.5 µM for 20h and processed for DRIP-seq. At *ARPC3*, *NCOA4 and SUZ12* loci, peaks increased near the promoter in *SF3B1*^{MUT} cells. Peaks at *HK1, NCOA4 and SUZ12* loci increased in control cells. At *SUZ12* locus, peaks in *SRSF2*^{MUT} or *NRAS*/bi*TET2*^{MUT} samples decreased. RPM: reads per million.

Supplementary Fig. 11: Effects of HDACi on splicing, clonal selection and proliferation.

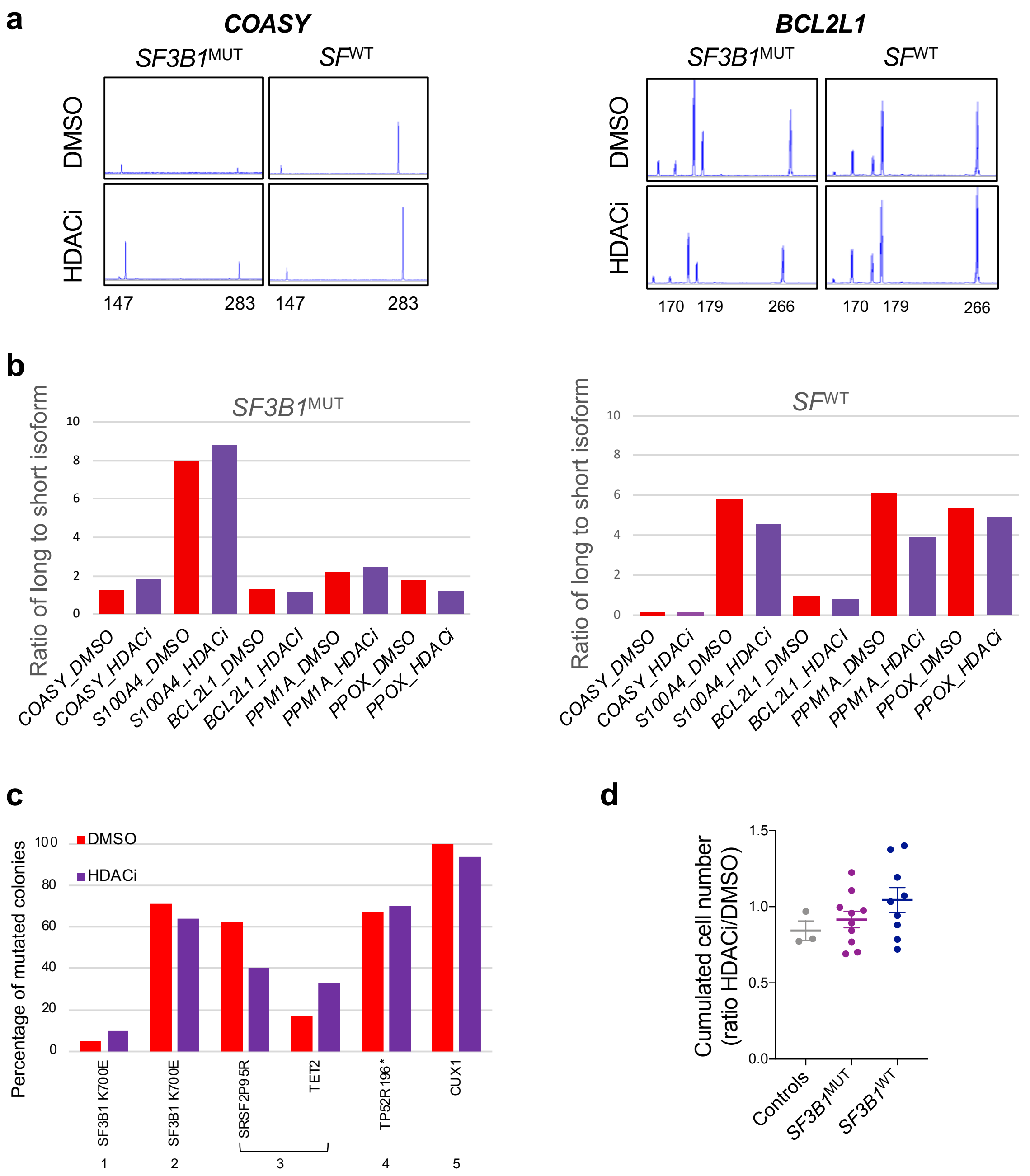

a,b. Fluorescent PCR fragment analysis of 2 genes *COASY* and *BCL2L1* with intron retention reduction in 1 *SF3B1*^{MUT} and 1 *SF*^{WT} basoE treated by HDACi 0.5 µM for 20h or DMSO. **a**. Chromatograms of representative experiments. **b.** Histograms showing the quantification of under the curve area expressed as ratios between the long and short fragments. **c.** Day 5-progenitors of 5 patients were seeded in methylcellulose at 5,000 cell/well. BFU-E were counted at d14 and picked (at least 20 colonies per sample and per condition) for single-colony PCR of the mutation identified at diagnosis. Results are expressed as percentages of mutated colonies**. d.** Erythroid cell amplification in 9 *SF3B1*^{MUT}, 8 *SF3B1*^{WT} and 3 controls expressed as ratio of cumulated cell number in HDACi to DMSO condition. Means ± standard error of the mean are indicated.

Supplementary Methods

Supplementary Methods 1: DRIP-sequencing products

Product	Characteristics	Reference produit	Supplier
S9.6 Ab	Anti-RNA:DNA Hybrid, 1mg/mL	ENH001	Kerafast
S9.6 Ab	mouse hybridoma	HB-8730	ATCC
RNaseH1	5,000 units/mL	M0297S	NEB
EcoRI	20,000 units/mL	R0101S	NEB
HindIII	20,000 units/mL	R0104S	NEB
BsrGI	10,000 units/mL	R0575S	NEB
XbaI	20,000 units/mL	R0145S	NEB
SspI	5,000 units/mL	R0132S	NEB
EDTA	0.5 M, pH 8, RNAse free	AM9260G	Thermo Fisher
TRIS	1 M, pH 8, RNase-free	AM9855G	Thermo Fisher
Sodium Dodecyl Sulfate	20% (WT/vol)	05030	Sigma-Aldrich
Proteinase K	20 mg/mL	03115828001	Roche
Spermidine	0.1 M	05292-1ML-F	Sigma-Aldrich
Bovine Serum Albumin	100X	B9001	NEB
Sodium chloride	NaCl 5 M	SX0420-5	Millipore
Agarose A/G beads		20421	Thermo Fischer
iScript reverse transcription supermix	RNase-Free	1708840	Bio-Rad
SsoAdvanced Universal SYBR Green Supermix	RNase-Free	1725272	Bio-Rad
Nuclease-free water		B1500S	NEB
Glycogen	RNase-Free	R0561	Thermo Fisher
Ethanol	absolute 200 proof, RNase free	V1001	VWR
Sodium phosphate	dibasic, anhydrous (HNa_2O_4P)	S374-500	Thermo Fisher
Sodium phosphate	monobasic (H_2NaO_4P)	S397-500	Thermo Fisher
Phenol/chloroform/isoamyl alcohol	1:24:1	75831-400ML	Thermo Fisher

Supplementary Methods 2: DRIP-qPCR primers

Gene	Name	Location	Forward (5' to 3')	Reverse (5' to 3')
RPL13A	Ribosomal protein L13A	3' of the gene	AGGTGCCTTGCTCACAGAGT	GGTTGCATTGCCCTCATTAC
TFPT	TCF fusion partner	5' of the gene	TCTGGGAGTCCAAGCAGACT	AAGGAGCCACTGAAGGGTTT
ABCC5_1	ATP-binding cassette subfamily C member 5 isoforms x1/1		GGCTGGCAGGTAAAAACAAA	TTTCACCGAACCTTGGTAGC
ABCC5_2	ATP-binding cassette subfamily C member 5 isoforms x1/1	intron 5 - 6	GAAGGCTGAAAGGACAGCAG	GGAGGCGCTGATGCTATTAC
IREB2	Iron-responsive element binding protein 2 isoforms 1/3	86bp	AGAGCACAAGCAGGAGGAAG	ATACATGGGTGGTCCTGGAA
TCIRG1	T cell immune regulator 1, vacuolar-type proton ATPase 116kda subunit a3 isoforms x1/a	84bp	CTGAGCTCCGACTCCTTGTC	GCTTGGGACTGTGGGAAGT
IFRD2	Interferon related developmental regulator 2		GACCAGGTCCTAGGAGCACA	ACATCCAGGTGAGGGGTCTT
TMX2	Thioredoxin related trans-membrane protein 2	82 bp	TGCCTTCCCTCTCACTGTTT	AGAGGGAGATGCCTTTGGTT
EGR1	Early growth response 1	downstream intergenic region	GAACGTTCAGCCTCGTTCTC	GGAAGGTGGAAGGAAACACA

Supplementary Methods 3: Flow cytometry antibodies and reagents

Species reactivity	Antibody	Fluorochrome	Clone and Immunoglobulin class	Reference	Dilution
**Human**	GPA (CD235a)	PE-Cy7	11E4B.76, IgG1 (mouse)	Beckman Coulter A71564	1/50
	CD71	FITC	YDJ1.2.2, IgG1 (mouse)	Beckman Coulter IM0483	1/50
	CD49d	APC	HP2/1, IgG1 (mouse)	Beckman Coulter B01682	1/50
	isotype control	PE-Cy7	679.1Mc7, IgG1 (mouse)	Beckman Coulter IM2475	1/50
	isotype control	FITC	679.1Mc7, IgG1 (mouse)	Beckman Coulter A07795	1/50
	Isotype control	APC	679.1Mc7, IgG1 (mouse)	Beckman Coulter A79393	1/50
**Mouse**	c-Kit	APC	2B8, IgG2b/κ (rat)	BD Biosciences 553356	1/250
	Ter119	PE	Ter-119, IgG2b/κ (rat)	BD Biosciences 553673	1/200
	isotype control	APC	A95-1, IgG2b/κ (rat)	BD Biosciences 553991	1/250
	isotype control	PE	A95-1, IgG2b/κ (rat)	BD Biosciences 553989	1/200

Supplementary Methods 4: BrdU labelling

Products	isotype	Fluorochrome	Reference	dilution
5-Bromo 2'deoxyuridine			BD Biosciences 559619	
Anti-BrdU antibody 3D4	IgG1κ (mouse)	FITC	BD Biosciences 556028	1/50
Anti-BrdU antibody 3D4	IgG1κ (mouse)	APC	BD Biosciences 51-23619L	1/50
RNase A			Macherey-Nagel 740505	
7-aminoactinomycin (7-AAD)			BD Biosciences 559925	

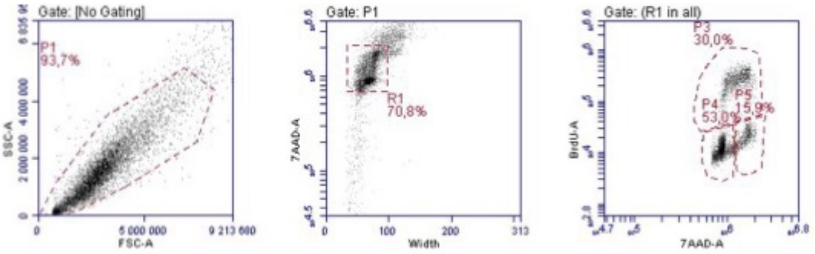

Supplementary Methods 5: DNA fiber combing antibodies and reagents

Antibody	Fluorochrome	Clone and Ig class	Reference	Dilution
anti-BrdU (anti-IdU)		B44, IgG1/κ (mouse)	347580, BD Biosciences	1/50
anti-BrdU (anti-CldU)		BU1/75, IgG2a/κ (rat)	ab6326, Abcam	1/50
Anti-mouse (anti-IdU)	Alexa 488	Polyclonal IgG (goat)	A11029, Thermo Fisher Scientific	1/50
Anti-rat (anti-CldU)	Alexa 555	Polyclonal IgG (goat)	A21434, Thermo Fisher Scientific	1/50
anti-ssDNA		16-19, IgG2a/κ (mouse)	MAB3034, Merck	1/25
Anti-mouse anti-ssDNA	Cy5*	Polyclonal IgG (goat)	A10524, Thermo Fisher Scientific	1/100
Anti-goat anti-ssDNA	Cy5	Polyclonal IgG (donkey)	ab6566, Abcam	1/100

Product	Reference
IdU	I7125, Merck
CldU	C6891, Merck
Thymidine	89270, Merck
Agarose	A4018, Merck
50-Well Disposable Plug Molds	1703713, Bio-Rad
Proteinase K	3115844001, Merck
β-agarase	M0392, New England BioLabs
2-(N-morpholino)-ethanosulfonic acid (MES)	M8250, Merck
Silane-treated coverslips	COV-002-RUO, Genomic Vision
Blocking solution	B10710, Thermo Fisher Scientific

Supplementary Methods 6: IF antibodies and reagents

Antibody	Fluorochrome	Characteristics	Reference	Dilution
p-RPA32, S33		Polyclonal IgG (rabbit)	Bethyl Laboratories A300-246A	1/500
p-RPA32, S4/8		Polyclonal IgG (rabbit)	Bethyl Laboratories A300-245A	1/250
γ-H2AX, S139		Polyclonal IgG (rabbit)	Cell Signaling Technology 25775	1/250
53BP1		Polyclonal IgG (rabbit)	Novus Biologicals NB100-904	1/500
Anti-rabbit	DyLight 488	Polyclonal IgG (donkey)	Bethyl Laboratories A120-108D2	1/200
DAPI		DNA staining	Thermo Fisher Scientific D1306	1/1000

Supplementary Methods 7: Primary antibodies for Western blot

Antibody	Characteristics	Reference	Dilution
β-actin	IgG1 (mouse)	A1978, Sigma Merck	1/10,000
p-RPA32 (S4/8)	Polyclonal IgG (rabbit)	A300-245A, Bethyl Laboratories	1/1,000
RPA32	12F3.3, IgG2a/κ (mouse)	GTX70243, GeneTex	1/1,000
p-CHK1 (S345)	Polyclonal IgG (rabbit)	2341, Cell Signaling Technology	1/1,000
CHK1	Polyclonal IgG (rabbit)	2345, Cell Signaling Technology	1/1,000
ECL-based detection system		10600002, VWR	

Supplementary Methods 8:

a. **Primers for RT-PCR, fluorescent RT-PCR and RT-qPCR**

Gene	**Forward**	**Reverse**
COASY	(6-FAM)- TTG ACC TTC CTC CGT TTC C	GCT GAA CTG AGT CGA AAT CC
S100A4	CTC TCT CCT CAG CGC TTC TT	ATA GCA ACA GCG TGT GCA AG
PPOX	TCC TAG TGG AGA GCA GTG AG	GCA CTT CTG AAT CCA AGC C
BCL2L1	(6-FAM) GGT GGG AGA TTC AGA GTC CA	CCA AAA CAC CTG CTC ACT CA
PPM1A	TGC TGT GAT GAG ACT CAG CTT	CTC CTT GGG TTC TGA GCA AT
HK1	CCAACATTCGTAAGGTCCATTCC	CCTCGGACTCCATGTGAACATT
BCL2L1	AACCAGCGGTTGAAGCGTTCCT	GCCACTTACCTGAATGACCACC
PPIA	CTCGAATAAGTTTGACTTGTGTTT	CTAGGCATGGGAGGGAACA
ACT	GTAGTTTCGTGGATGCCACAG	GAGCTACGAGCTGCCTGACG

b. **Primers for PCR on colonies**

Gene	*Forward*	*Reverse*
SF3B1	TGTTGGGGCATAGTTAAAACCT	TGTTAGAACCATGAAACATATCCA
SRSF2	GGCCGCCACTCAGAGCTA	CGCGGACCTTTGTGAGGT
TET2	GACCAATGTCAGAACACCTCAA	TTGATTTTGAATACTGATTTTCACCA
TP53	AAGCTCCTGAGGTGTAGACGC	GGGCCAGACCTAAGAGCAATC
CUX1	CTAGGGCCGACGAGATTGAA	ACTTCGGGTGACGCATCTTC
