# Supplementary material for: Accelerated DNA replication fork speed due to loss of R-loops in myelodysplastic syndromes with SF3B1 mutation
Source: Nat Commun. 2024 Apr 8;15:3016. doi: 10.1038/s41467-024-46547-7 (PMC11001894; doi:10.1038/s41467-024-46547-7)
Supplement: Supplementary file 3 — Description of Additional Supplementary Files [file 41467_2024_46547_MOESM3_ESM.pdf]

Supplementary Data

Supplementary Data 1:

RNA-sequencing data analysis of bone marrow mononuclear cell samples from 27 low-risk MDS patients: Differential gene expression and splicing events

Supplementary Data 2:

RNA-sequencing data analysis of human basophilic erythroblasts and polychromatophilic erythroblasts: differential gene expression and splicing events

Supplementary Data 3:

Proteomic data analysis of human basophilic erythroblasts and polychromatophilic erythroblasts.

Supplementary Data 4:

DRIP-sequencing data analysis of human basophilic erythroblasts

Supplementary Data 5:

RNA-sequencing and proteomic data analyses of mouse G1E-ER4 erythroblasts

Supplementary Data 6:

DRIP-sequencing data analysis of human basophilic erythroblasts treated or not with histone deacetylase inhibitor vorinostat.

Supplementary Data 7:

Gene sets of DNA repair and cell cycle regulatory pathways
