# Supplementary material for: Accelerated DNA replication fork speed due to loss of R-loops in myelodysplastic syndromes with SF3B1 mutation
Source: Nat Commun. 2024 Apr 8;15:3016. doi: 10.1038/s41467-024-46547-7 (PMC11001894; doi:10.1038/s41467-024-46547-7)
Supplement: Supplementary file 11 — Reporting Summary [file 41467_2024_46547_MOESM11_ESM.pdf]

nature portfolio

Corresponding author(s): Michaëla Fontenay

Last updated by author(s): Feb 22, 2024

Reporting Summary

Nature Portfolio wishes to improve the reproducibility of the work that we publish. This form provides structure for consistency and transparency in reporting. For further information on Nature Portfolio policies, see our Editorial Policies and the Editorial Policy Checklist.

Statistics

For all statistical analyses, confirm that the following items are present in the figure legend, table legend, main text, or Methods section.

n/a	Confirmed	
☐	☒	The exact sample size (*n*) for each experimental group/condition, given as a discrete number and unit of measurement
☐	☒	A statement on whether measurements were taken from distinct samples or whether the same sample was measured repeatedly
☐	☒	The statistical test(s) used AND whether they are one- or two-sided<br>*Only common tests should be described solely by name; describe more complex techniques in the Methods section.*
☒	☐	A description of all covariates tested
☐	☒	A description of any assumptions or corrections, such as tests of normality and adjustment for multiple comparisons
☐	☒	A full description of the statistical parameters including central tendency (e.g. means) or other basic estimates (e.g. regression coefficient) AND variation (e.g. standard deviation) or associated estimates of uncertainty (e.g. confidence intervals)
☐	☒	For null hypothesis testing, the test statistic (e.g. *F*, *t*, *r*) with confidence intervals, effect sizes, degrees of freedom and *P* value noted<br>*Give P values as exact values whenever suitable.*
☒	☐	For Bayesian analysis, information on the choice of priors and Markov chain Monte Carlo settings
☒	☐	For hierarchical and complex designs, identification of the appropriate level for tests and full reporting of outcomes
☒	☐	Estimates of effect sizes (e.g. Cohen's *d*, Pearson's *r*), indicating how they were calculated

Our web collection on statistics for biologists contains articles on many of the points above.

Software and code

Policy information about availability of computer code

Data collection

No software was used.

Data analysis

Open-source softwares used in the study :

RNA-seq and DRIP-seq :
Reads were trimmed and filtered with fastp (v0.23.4).
samblaster (v0.1.24) to remove duplicated reads.
samtools (v1.10) for sorting and indexing BAM files.
bedtools (v2.27.1) was used for overlap analyses.

RNA-seq :
Mapping and counting using STAR (v2.7.9) to align the reads against the human reference genome GRCh37 (UCSC version hg19) (https://www.gencodegenes.org/)
Read count normalizations and groups comparisons were performed using DESeq2 (v1.30.1)
Differentially expressed splicing events using KisSplice (v2.6.2), KisSplice2refgenome (v2.0.7) and KissDE (v1.15.3)

DRIP-seq :
Mapping using Bowtie2 (v2.3.5.1) to the human reference genome GRCh37 (UCSC version hg19) (https://www.gencodegenes.org/)
Peaks calling for each replicate with MACS3 (v3.0.0)
Called peaks were merged by groups of biological replicates with MSPC (v5.5.0)
featureCounts (v2.0.0), to count reads on restriction fragments with significant peaks and differential expression analysis with DESeq2

(v1.30.1)

Proteomics
Mass spectrometry data analyzed using Maxquant (v2.1.1.0)
Cytoscape (v3.9.1) for pathway visualization.

Commercial softwares used in the study :
Ingenuity Pathway Analysis (v76765 844) (QIAGEN Inc., https://wwuw.giagenbioinformatics.com/products/ingenuitypathway-analysis)

For manuscripts utilizing custom algorithms or software that are central to the research but not yet described in published literature, software must be made available to editors and reviewers. We strongly encourage code deposition in a community repository (e.g. GitHub). See the Nature Portfolio guidelines for submitting code & software for further information.

Data

Policy information about availability of data

All manuscripts must include a data availability statement. This statement should provide the following information, where applicable:

- Accession codes, unique identifiers, or web links for publicly available datasets
- A description of any restrictions on data availability
- For clinical datasets or third party data, please ensure that the statement adheres to our policy

Data availability

Reference genome and annotations are available on Gencode :
https://www.gencodegenes.org/human/release_19.html

Data are available on NCBI's Gene Expression Omnibus (GEO) for RNA-seq and DRIP-seq data :

RNA-seq BM MNC cohort of 27 cases (suppl Table 1): GSE220525
https://www.ncbi.nlm.nih.gov/geo/query/acc.cgi?acc=GSE220525

RNA-seq of human basophilic erythroblasts and polychromatophilic erythroblasts (suppl Table 2) : GSE220523
https://www.ncbi.nlm.nih.gov/geo/query/acc.cgi?acc=GSE220523

DRIP-seq of human basophilic erythroblasts (suppl Table 4): GSE220271
https://www.ncbi.nlm.nih.gov/geo/query/acc.cgi?acc=GSE220271

RNA-seq of mouse G1EER erythroblasts (suppl Table 5): GSE220516
https://www.ncbi.nlm.nih.gov/geo/query/acc.cgi?acc=GSE220516

RNA-seq BM MNC cohort of 189 (suppl Fig 1): GSE220518
Declare that the RNA-seq data of MDS-RIGHT cohort are part of an on-going multi-omics study implicating that these data will remain private or available upon request to david.rombaut@aphp.fr until the manuscript is accepted for publication.

Data are available on ProteomeXchange Consortium via the PRIDE partner repository for proteomics data :

Human erythroblast proteome (Suppl Table 3) and mouse erythroblast proteome (Suppl Table 6) : PXD038700
https://www.ebi.ac.uk/pride/archive/projects/PXD038700

Research involving human participants, their data, or biological material

Policy information about studies with human participants or human data. See also policy information about sex, gender (identity/presentation), and sexual orientation and race, ethnicity and racism.

Reporting on sex and gender	We used the term sex (biological attribute) to describe the studied cohort of patients (Table 1)
Reporting on race, ethnicity, or other socially relevant groupings	Not applicable.
Population characteristics	The covariates are age, sex, disease WHO category, hemogram parameters, bone marrow parameters, karyotypic abnormalities, and genotypic abnormalities including mutations in SF381 gene (Table 1)
Recruitment	Patients with lower-risk MDS were consecutively enrolled in the three hospitals (Cochin, Saint-Louis and Nice) and classified into two groups: MDS with SF3B1 mutation and MDS without SF3B1 mutation.<br>The cohort of 189 LR-MDS patients was enrolled consecutively between 2010 and 2015 by French investigation centres, at diagnosis, before any disease-modifying treatments.
Ethics oversight	Each patient provided his/her consent to the use of bone marrow samples and clinical data for this non-interventional research which was approved by institutional review board (IRB) and ethics committee (IR8 numbers: IdFV 212-A01395-38 EudraCT 2012-002990-7338; OncoCCH 2015-08-11-DC).<br>French cohort of 189 LR-MDS patients: IRB IdF X: 2010-A00033-36-2753

Note that full information on the approval of the study protocol must also be provided in the manuscript.

Field-specific reporting

Please select the one below that is the best fit for your research. If you are not sure, read the appropriate sections before making your selection.

☒ Life sciences ☐ Behavioural & social sciences ☐ Ecological, evolutionary & environmental sciences

For a reference copy of the document with all sections, see nature.com/documents/nr-reporting-summary-flat.pdf

Life sciences study design

All studies must disclose on these points even when the disclosure is negative.

Sample size	Sample sizes were chosen to provide at least three biologically independent samples for each condition in all the experiments, to ensure precision and robustness.
Data exclusions	RNA-seq or DRIP-seq data were excluded from analyses when quality control criterias were not met.
Replication	To assess the reproducibility of the findings,<br>- at least three biologically independent samples in each catergory were studied in experiments using patient-derived primary samples, except controls for p-RPA32 s33 IF experiments (n=2)<br>- and at least three independent experiments using erythroblatic cell lines were performed<br>Numbers of primary biologically independent samples or cell line replicates of the experiments are indicated in the figure legends.
Randomization	Allocation was not random. The presence of a mutation in SF3B1 gene was the distinctive parameter between the patient groups of comparison. SF3B1-mutated MDS patient samples were compared to SF3B1-unmutated MDS patient samples, and to healthy, age matched controls. Covariates (age, sex, karyotypes and revised-International Prognosis Scoring System were balanced between the two groups of MDS patients. As expected from the literature, the SF3B1-mutated group had lower Hb level, lower bone marrow blast percentage, higher bone marrow erythroblast percentage, higher ringed sideroblast percentage (Table 1).<br>When cell lines were used, allocation was not random. CRISPR-Cas9 Sf3b1 K700E mutant cell line was compared to CRISPR-Cas9 K700K wild-type cell line.
Blinding	Omics and functional studies are performed to describe the consequences of a mutation. Annotation of samples was performed according to the mutational status, to allow performing comparative analysis of quantitative data.<br>For qualitative data, cytological examination was performed blindly, by two independent cytologists.

Reporting for specific materials, systems and methods

We require information from authors about some types of materials, experimental systems and methods used in many studies. Here, indicate whether each material, system or method listed is relevant to your study. If you are not sure if a list item applies to your research, read the appropriate section before selecting a response.

Materials & experimental systems

n/a	Involved in the study	
☐	☒	Antibodies
☐	☒	Eukaryotic cell lines
☒	☐	Palaeontology and archaeology
☒	☐	Animals and other organisms
☐	☒	Clinical data
☒	☐	Dual use research of concern
☒	☐	Plants

Methods

n/a	Involved in the study	
☐	☒	ChIP-seq
☐	☒	Flow cytometry
☒	☐	MRI-based neuroimaging

Antibodies

Antibodies used	Antibodies Fluorochrome, isotype Clone/ Supplier/ Catalog number / Batch number<br>anti-GPA (CD235a) PE-Cy7, IgG1 (mouse) 11E4B.76 / Beckman Coulter/ A71564 / 200060<br>CD71 FITC, IgG1 (mouse) YDJ1.2.2/ Beckman Coulter / IM0483 / 59<br>isotypic control PE-Cy7, IgG1 (mouse) 679.1Mc7/ Beckman Coulter / IM2475 / 22<br>isotypic control FITC, IgG1 (mouse) 679.1Mc7/ Beckman Coulter / A07795 / 12<br>anti-mouse c-Kit APC, IgG2b/κ (rat) 2B8 / BD Biosciences / 553356 / 23<br>anti-mouse Ter119 PE, IgG2b/κ (rat) Ter-119, BD Biosciences / 553673 / B328361<br>isotypic control APC, IgG2b/κ (rat) A95-1/ BD Biosciences / 553991 / 61696<br>isotypic control PE, IgG2b/κ (rat) A95-1/ BD Biosciences / 553989 / 53124<br>anti-DNA:RNA hybrid IgG2a (mouse) S9.6 / Kerafast / ENH001 / 200826<br>anti-DNA:RNA hybrid IgG2a (mouse) S9.6/ Monoclonal hybridoma / ATCC HB-8730

anti-BrdU FITC, 1gG1/κ (mouse) 3D4/ BD Biosciences / 556028 / 7174675
anti-BrdU APC, 1gG1/κ (mouse) 3D4/ BD Biosciences 51-23619L / 8207809
anti-BrdU (anti-IdU) IgG1/κ (mouse) B44 / BD Biosciences 347580 / 0029341 & 3016583
anti-BrdU (anti-CldU) IgG2a/κ (rat) BU1/75/ Abcam ab6326 / 1009715-12
anti-mouse IgG (H+L) Alexa 488, Polyclonal IgG (goat) / Thermo Fisher Scientific A11029 / 2179204 & 1789729
anti-rat IgG (H+L) Alexa 555, Polyclonal IgG (goat) / Thermo Fisher Scientific / A21434 / 2147636 & 1987272
anti-ssDNA IgG2a/k (mouse) 16-19/ Merck MAB3034 / 2684913 & 2736584
anti-p-RPA32 s33, polyclonal IgG (rabbit) / Bethyl Laboratories / A300-246A / 7

anti-mouse IgG (H+L) Cy5*, Polyclonal IgG (goat) / Thermo Fisher Scientific / A10524 / 2155288
anti-goat IgG (H+L) Cy5, Polyclonal IgG (donkey) / Abcam / ab 6566 / GR3203761-22 & 1029671-3
p-RPA32, S4/8 Polyclonal IgG (rabbit) / Bethyl Laboratories / A300-245A / M8
γ-H2AX, S139 Polyclonal IgG (rabbit) / Cell Signaling Technology 25775 / 11
53BP1 Polyclonal IgG (rabbit) / Novus Biologicals / NB100-904 / 100814
anti-rabbit IgG (H+L) DyLight 488, Polyclonal IgG (donkey) / Bethyl Laboratories / A120108-D2 / 7
β-actin IgG1 (mouse) / Merck, Sigma Aldrich / A1978 / 0000086303
RPA32 IgG2a/κ (mouse) 12F3.3/ GeneTex / GTX70243 / 40004
p-CHK1 (S345) Polyclonal IgG (rabbit) / Cell Signaling Technology 241 / 18
CHK1 Polyclonal IgG (rabbit) / Cell Signaling Technology / 2345 / 17

Validation

clone S9.6 Ab (Kerafast) recognizes DNA-RNA hybrid, but not double-stranded DNA (dsDNA)
validation for DRIP-seq in Ginno, P.A., et al. (2012). Mol. Cell, 45(6):814-825)
and in Sanz and Chédin Nat Protocols 2019 Jun;14(6):1734-1755

clone 16-19 anti-ssDNA : validation for DNA combing experiments in
Letessier A, Millot GA, Koundrioukoff S, Lachagès AM, Vogt N, Hansen RS, Malfoy B, Brison O, Debatisse M. Cell-type-specific replication initiation programs set fragility of the FRA3B fragile site. Nature. 2011 Feb 3;470(7332):120-3.
and in Fu, H; Martin, MM; Regairaz, M; Huang, L; You, Y; Lin, CM; Ryan, M; Kim, R; Shimura, T; Pommier, Y; Aladjem, MI. Nature communications 6: 6746, 2015

clone 3D4 mouse anti-BrdU: validation for BrdU assay in
Beisker W, Dolbeare F, Gray JW. An improved immunocytochemical procedure for high-sensitivity detection of incorporated bromodeoxyuridine. Cytometry. 1987; 8:235

clone B44, 347580 BD Biosciences, mouse anti-BrdU: (IdU) : validation for DNA combing experiment in
Letessier A, Millot GA, Koundrioukoff S, Lachagès AM, Vogt N, Hansen RS, Malfoy B, Brison O, Debatisse M. Cell-type-specific replication initiation programs set fragility of the FRA3B fragile site. Nature. 2011 Feb 3;470(7332):120-3.

clone BU1/ab6326 Abcam: anti-BrdU (CldU): validation for DNA combing experiment in
Okamoto Y et al. SLFN11 promotes stalled fork degradation that underlies the phenotype in Fanconi anemia cells. Blood 137:336-348 (2021)

γ-H2AX, S139 Polyclonal IgG (rabbit) 25775 Cell Signaling Technology: validation for IF and Western blot
Shorrocks A-M.K. et al Nat Commun. 2021 Jan 26;12(1):585.

polyclonal anti-pRPA32 s4/8 A300-245A Bethyl Laboratories: validation for IF and Western blot
https://fortis-datasheets.s3.us-east-2.amazonaws.com/A300-245A-5.pdf

polyclonal anti-pRPA32 s33 IgG (rabbit) A300-246A Bethyl Laboratories: validation for IF in
Oo J et al. Long non-coding RNA PCAT19 safeguards DNA in quiescent endothelial cells by preventing uncontrolled ^hosphorylation of RPA2. Cell Rep 2022; 41: 111670.

53BP1 Polyclonal IgG (rabbit) NB100-904 Novus Biologicals: vlidation for IF in
Beli P, Mosler T, Conte F et al. R-loop proximity proteomics identifies a role of DDX41 in transcription-associated genomic instability Nat Commun Dec 17 2021 [PMID: 34916496]

RPA32 IgG2a/κ (mouse) 12F3.3/GTX70243 GeneTex: validation for western blot in
Yoshihara T et al. EMBO J 2004; 23 (3):670-80 XRCC3 deficiency results in a defect in recombination and increased endoreduplication in human cells.

p-CHK1 (S345) Polyclonal IgG (rabbit) 2341 Cell Signaling Technology: validation for IF and Western blot
Foskolou IP et al. Ribonucleotide Reductase Requires Subunit Switching in Hypoxia to Maintain DNA Replication. Mol Cell 2017

CHK1 Polyclonal IgG (rabbit) 2345 Cell Signaling Technology: Kotsantis P et al. Increased global transcription activity as a mechanism of replication stress in cancer. Nat Commun 2016; 7 : 13087

Eukaryotic cell lines

Policy information about cell lines and Sex and Gender in Research

Cell line source(s)

The murine male G1E-ER4 cell line expressing a GATA1-estrogen receptor fusion gene (1) was used to edit mutant Sf3b1K700E and isogenic Sf3b1WT using CRISPR-Cas9 strategy (2)

1. Weiss, M. J., Yu, C. & Orkin, S. H. Erythroid-cell-specific properties of transcription factor GATA-1 revealed by

phenotypic rescue of a gene-targeted cell line. Mol. Cell. Biol. 17, 1642–1651 (1997).

2. Bondu, S. et al. A variant erythroferrone disrupts iron homeostasis in SF3B1-mutated myelodysplastic syndrome. Sci Transl Med 11, eaav5467 (2019).

Authentication	Karyotype of clone G1E-ER4 was performed before CRISPR-Cas9 procedure. Differentiation testing upon estradiol
Mycoplasma contamination	I confirm that G1E-ER4 cell lines were tested and found negative for mycoplasma contamination
Commonly misidentified lines (See ICLAC register)	There is no commonly misidentified cell line in this study

Clinical data

Policy information about clinical studies

All manuscripts should comply with the ICMJE guidelines for publication of clinical research and a completed CONSORT checklist must be included with all submissions.

Clinical trial registration	This study is not a clinical trial
Study protocol	A full trial protocol is not available because this study is not a clinical trial
Data collection	Annotations of each primary sample was performed at inclusion and updated at the end of the study.
Outcomes	There was no outcome measurements for this biological study

Plants

Seed stocks	Not applicable
Novel plant genotypes	Not applicable
Authentication	Not applicable

ChIP-seq

Data deposition

☒ Confirm that both raw and final processed data have been deposited in a public database such as GEO.

☒ Confirm that you have deposited or provided access to graph files (e.g. BED files) for the called peaks.

Data access links *May remain private before publication.*	Data are NOT ChIP-seq, but DRIP-seq of human basophilic erythroblasts (suppl Table 4): GSE220271<br>https://www.ncbi.nlm.nih.gov/geo/query/acc.cgi?acc=GSE220271
Files in database submission	76*2 fastq files, plus 52 bedGraph files, on https://www.ncbi.nlm.nih.gov/geo/query/acc.cgi?acc=GSE220271
Genome browser session (e.g. UCSC)	There is no on line session for this study

Methodology

Replicates	Each biological replicate consisted in a culture of erythroblasts deriving from the bone marrow progenitors of one patient<br>To assess immunoprecipitation specificity, each biological replicate was splitted in two conditions without or with RNase H1 treatment that disrupts R-loops.<br>Number of replicates: 15 from 15 distinct patients or controls<br>Replicate agreement: Analysis of differential peaks using DeSeq2 to obtain a Principal Component Analysis allowing a comparison between samples
Sequencing depth	Reads length is 100bp, paired-end, for 52 samples, each samples being treated with RNAse H1 or not.<br><br>Mean reads number for samples not treated with RNAse H1 is 63M (36M standard deviation)<br>Mean reads number for samples treated with RNAse H1 is 58M (30.6M standard deviation)<br><br>Mean uniquely mapped reads for samples not treated with RNAse H1 is 12.4M (8.8M standard deviation)

Mean uniquely mapped reads for samples treated with RNAse H1 is 11.2M (11.5M standard deviation)

Antibodies

anti-DNA:RNA hybrid IgG2a (mouse) S9.6/ENH001 Kerafast

Peak calling parameters

Mapping (indexing and mapping)

```
bowtie2-build GRCh37.p13.genome.fa \
   bowtieIDX_drip/GRCh37 \
   --threads 64


bowtie2 \
   -x bowtieIDX_drip/GRCh37 \
   -p 64 \
   --no-discordant --no-mixed \
   -1 ${FQDIR}/${fastq} \
   -2 ${FQDIR}/${fastq2} | \
   samblaster -r | \
   samtools view -q 10 -b -@ 64 - | \
   samtools sort -@ 64 -o ${BAM}/${output}/${output}.bam -
```

Calling

```
macs3 callpeak --broad -broad-cutoff 0.05 -g hs \
   -t Soutput/Soutput.bam -f BAMPE \
   -c $rh/$rh.bam Sctrl/$input.bam --scale-to small \
   -n Soutput --outdir Soutput/broad05
```

Data quality

DRIP-seq quality was assessed with FastQC, and fastp was used for trimming. Trimming steps included a trimming of polyG tail (meaning no signal with NovaSeq) as well as a low complexity filter (set at the default 30%). Citation from fastp github (https://github.com/OpenGene/fastp#low-complexity-filter) :

"The complexity is defined as the percentage of base that is different from its next base (base[i] = base[i+1]). For example:
a 51-bp sequence, with 3 bases that is different from its next base
seq = 'AAAATTTTTTTTTTTTTTTTTTTTTGGGGGGGGGGGGGGGGGGGGGGCCCC'
complexity = 3/(51-1) = 6%"

```
fastp -w 16 -i $fastq -1 $fastq2 -o trim/Soutput"_R1.fastq" -O trim/Soutput"_R2.fastq" \
   --detect_adapter_for_pe\
   --trim_front1 1 --trim_front2 1 \
   --trim_poly_g \
   --trim_poly_x \
   --low_complexity_filter
```

To ensure DRIP specificity, peak calling for each sample was realized with their input control as well as the same sample treated with RNAse H1.

Shared peaks (MSPC, weak threshold 1e-4, stringency threshold 1e-8, gamma threshold 1e-8, adjusted p-value 0.05) from biological replicates after calling (MACS3, q-value 0.10) :

MUT
1_NT : 216
2_NT : 197
3_NT : 199
3_HDACi : 2064
4_NT : 87
4_HDACi : 67
5_NT : 236
5_HDACi : 1869

WT
1_NT : 5799
2_NT : 6618
3_NT : 1618
4_NT : 2127
4_HDACI : 175
5_NT : 1953
5_HDACi : 154
6_NT : 1528
6_HDACi : 106

Controls
1_NT : 1692
1_HDACi : 1959
2_NT : 2204

	2_HDACi : 2218<br>3_NT : 226<br>3_HDACi : 35<br>4_NT : 1619<br>4_HDACi : 2842
Software	Reads mapping to the human reference genome (GENCODE, GRCh37): Bowtie2<br>Peak calling: MACS algorithm (MACS3)<br>For merging by groups of biological replicates and perform the analysis of shared peaks, we used MSPC. (Fisher test and multiple testing correction with Benjamini Hochberg procedure to assess the significance of overlapping peaks).

Flow Cytometry

Plots

Confirm that:

☒ The axis labels state the marker and fluorochrome used (e.g. CD4-FITC).

☒ The axis scales are clearly visible. Include numbers along axes only for bottom left plot of group (a 'group' is an analysis of identical markers).

☒ All plots are contour plots with outliers or pseudocolor plots.

☒ A numerical value for number of cells or percentage (with statistics) is provided.

Methodology

Sample preparation	Primary erythroblasts derived in culture from CD34+ bone marrow progenitors or mouse erythroblastic cell lines were harvested, washed in PBS before labelling with fluorescent antibodies.
Instrument	LSRFortessa apparatus (BD Biosciences) for erythroid differentiation follow-up or BD Accuri C6 flow cytometer for BrdU incorporation measurement
Software	Kaluza software (Beckman Coulter) for erythroid differentiation or CFlow Plus software (BD Biosciences) for BrdU incorporation measurement
Cell population abundance	No cell sorting was performed using an automatic cell sorter. CD34+ bone marrow progenitors were sorted on Milteny columns and the purity was assessed by CD34 labeling using a distinct CD34 antibody.
Gating strategy	FSC/SSC gates were used to identify viable cells and exclude doublets.<br>Gating on viable cells was used to measure differentiation or BrdU incorporation according to the manufactorer instructions (https://www.bdbiosciences.com/content/dam/bdb/products/global/reagents/flow-cytometry-reagents/research-reagents/panels-multicolor-cocktails-ruo/559619_base/pdf/23-12721.pdf).

☒ Tick this box to confirm that a figure exemplifying the gating strategy is provided in the Supplementary Information.
